# *Escherichia coli* strains possessing a four amino acid YRIN insertion in PBP3 identified as part of the SIDERO-WT-2014 surveillance study

**DOI:** 10.1093/jacamr/dlaa081

**Published:** 2020-10-05

**Authors:** Takafumi Sato, Akinobu Ito, Yoshino Ishioka, Shuhei Matsumoto, Masatomo Rokushima, Krystyna M Kazmierczak, Meredith Hackel, Daniel F Sahm, Yoshinori Yamano

**Affiliations:** 1 Drug Discovery & Diseases Research Laboratory, Shionogi & Co., Ltd., Osaka, Japan; 2 Drug Efficacy Evaluation I, Shionogi TechnoAdvance Research & Co., Ltd., Osaka Japan; 3 International Health Management Associates, Inc., Schaumburg, IL, USA

## Abstract

**Background:**

In addition to carbapenemases, dissemination of recently reported *Escherichia coli* lineages possessing a four amino acid insertion in PBP3 (encoded by *ftsI*) that confers reduced susceptibility to PBP3-targeted β-lactams, such as ceftazidime, can pose a threat of antimicrobial resistance.

**Objectives:**

To evaluate genotypic and phenotypic characteristics of *E. coli* possessing the mutated PBP3 collected during SIDERO-WT-2014 surveillance.

**Methods:**

A subset of 65 *E. coli* clinical isolates with MICs ≥2 mg/L for ceftazidime/avibactam, ceftolozane/tazobactam or cefiderocol, among a total of 1529 isolates from the multinational surveillance study, were subjected to gene analysis and antimicrobial susceptibility testing. Isogenic PBP3 mutants were constructed to confirm experimentally an impact on antimicrobial susceptibility.

**Results:**

Eleven strains possessing a YRIN-inserted PBP3 were identified, consisting of nine strains collected from the same hospital in Turkey (ST1284) and one each from the USA and Italy (ST361). Strains associated with each ST lineage possessed similar genetic backgrounds including β-lactamase genotypes; all nine strains from Turkey carried CMY-42, OXA-1 and the OXA-181 carbapenemase (five strains additionally carried CTX-M-15 ESBL), whereas the two other strains carried CMY-42 and TEM-1, indicating dissemination driven by selective pressure. The presence of the YRIN insertion contributed to reduced susceptibility to aztreonam, ceftazidime, cefepime and ceftolozane/tazobactam, although the strains remained susceptible to ceftazidime/avibactam despite relatively high MICs.

**Conclusions:**

*E. coli* strains of both ST1284 and ST361 lineages, possessing YRIN-inserted PBP3, are disseminating in several regions. The YRIN insertion in PBP3 occurred with multiple β-lactamases, which indicates frequent cross-resistance to other β-lactams.

## Introduction

Unusual PBP3 substitutions, consisting of a four amino acid insertion of YRIN or YRIK after position P333 or TIPY after position Y334, have been reported in *Escherichia coli* clinical isolates.^1^^,^^2^ These altered PBP3 proteins conferred reduced susceptibility to a broad range of β-lactams, such as ceftazidime, cefepime and aztreonam, due to the decreased accessibility of the β-lactams to the transpeptidase pocket of PBP3. The YRIN(K) insertion in PBP3 was first identified in nine MLST lineages of *E. coli*, including ST101, ST405 and ST410, isolated in Asian and Middle Eastern countries, especially India, and was enriched among NDM-type carbapenemase-carrying strains.^1^ In a recent report, the four amino acid insertion in PBP3 was observed in several *E. coli* lineages according to NCBI database analysis, and the presence of this mutation as well as mutations in *ompC* and *ompF* was associated with acquisition of carbapenemase genes. It is still unknown which lineages carrying YRIN(K)-inserted PBP3 are widespread or limited in geographic distribution and which have clinical importance.^3^

Here, we evaluated the genotypic and phenotypic characteristics of *E. coli* strains possessing four amino acid insertions in PBP3 by re-analysing isolates collected as part of the SIDERO-WT-2014 surveillance study.^4^

## Materials and methods

### Screening for amino acid insertion in PBP3

The susceptibility data from 1529 *E. coli* isolates reported for SIDERO-WT-2014, the first multinational surveillance programme (North America and 13 European countries) conducted in support of the siderophore cephalosporin cefiderocol by International Health Management Associates, Inc. (IHMA; Schaumburg, IL, USA), were used.^4^ MICs ≥2(/4) mg/L for ceftazidime/avibactam and ≥4(/4) mg/L for aztreonam/avibactam have been reported for strains possessing YRIN(K)- and TYPI-inserted PBP3.^1^^,^^2^ Because aztreonam/avibactam was not studied as part of SIDERO-WT-2014, the MIC criterion of ≥2 mg/L was applied to ceftazidime/avibactam and two other PBP3-targeted cephalosporins tested in the surveillance study, ceftolozane/tazobactam and cefiderocol, to select strains for *ftsI* (PBP3) gene sequencing.

### Analyses of ftsI, MLST, β-lactamases and SNPs

Sanger sequencing and/or WGS by the Illumina MiSeq system (San Diego, CA, USA) were conducted as described in the Supplementary data (available as Supplementary data at *JAC* Online). The sequences of *ftsI* and the translated PBP3 protein from WT *E. coli* K-12 (GenBank accession number NC_000913) were used as references for analysis. MLST profiles were determined by comparison of seven allele sequences in the public database (http://mlst.warwick.ac.uk/mlst/dbs/Ecoli).^5^ β-Lactamase sequences were detected using the WU-blast 2.0 algorithm (http://genetics.bwh.harvard.edu/msblast/) and the ResFinder database (https://cge.cbs.dtu.dk/services/ResFinder/) using Genedata Selector 5.2.3 (Genedata, Switzerland). For SNP analysis, the trimmed reads were mapped against the *E. coli* K-12 genome and variant calls were performed using Microbial Genomics Module 3.5.1 in CLC Gx.

### Antibacterials and susceptibility testing

Cefiderocol, ceftolozane and avibactam were synthesized at Shionogi & Co., Ltd (Osaka, Japan) and other tested antibacterials were obtained commercially. MICs were determined by broth microdilution according to CLSI guidelines.^6^^,^^7^ To test cefiderocol, the CLSI-approved methodology using iron-depleted CAMHB (ID-CAMHB) was applied.^6^ Isogenic PBP3 mutants possessing a YRIN or YRIK insertion were constructed from *E. coli* MG1655 (K-12 derivative) by chromosomal recombination.^1^^,^^8^

## Results

### Substitutions and amino acid insertions in PBP3

A total of 65 isolates with MIC ≥2 mg/L for one or more of three PBP3-targeted cephalosporins were selected for analysis of *ftsI* gene sequences. Eleven strains (9 strains from Turkey and 1 each from Italy and the USA) encoded a PBP3 protein possessing the YRIN insertion after amino acid P333 and the amino acid substitution I532L; two or three additional substitutions were found in two strains (Table 1 and Table S1; additional substitutions in other strains). The SNPs E349K and I532L were previously reported in association with the YRIN insertion in PBP3, and these *ftsI* alleles are suggested to be disseminated by horizontal gene transfer.^3^ The nine strains were collected from only one hospital in Turkey, although five medical institutions in that country participated in the SIDERO-WT-2014 surveillance programme (Table S2). Isolates with PBP3 possessing the YRIK or TIPY insertion were not found in this study.

**Table 1.** Genotype and phenotype information of *E. coli* strains possessing a YRIN insertion in PBP3

Strain	Source	MLST	Substitutions and insertion in PBP3^a^	β-Lactamases	Antibacterials/MIC (mg/L)								
					ATM	ATM/AVI	CAZ/AVI	COZ/TAZ	CFD	FEP	MEM	CIP	CST
SR202162	Italy	361	YRIN, Q227H, E349K, I532L, N533D	CMY-42, TEM-1	32	4	2	>64	0.5	8	≤0.06	>8	0.5
SR202172	USA	361	YRIN, Q227H, E349K, I532L	CMY-42, TEM-1	64	4	2	64	0.25	8	0.12	>8	0.5
SR202163	Turkey	1284	YRIN, I532L	CMY-42, OXA-1, OXA-181	32	4	1	>64	2	8	0.5	>8	1
SR202167	Turkey	1284	YRIN, I532L	CMY-42, OXA-1, OXA-181	32	4	1	>64	2	8	0.25	>8	≤0.25
SR202168	Turkey	1284	YRIN, I532L	CMY-42, OXA-1, OXA-181	32	4	1	>64	1	4	0.5	>8	0.5
SR202169	Turkey	1284	YRIN, I532L	CMY-42, OXA-1, OXA-181	32	4	2	>64	1	8	0.5	>8	0.5
SR202164	Turkey	1284	YRIN, I532L	CMY-42, OXA-1, OXA-181, CTX-M-15	>64	4	1	>64	4	>64	1	>8	0.5
SR202165	Turkey	1284	YRIN, I532L	CMY-42, OXA-1, OXA-181, CTX-M-15	>64	4	2	>64	2	>64	0.5	>8	0.5
SR202166	Turkey	1284	YRIN, I532L	CMY-42, OXA-1, OXA-181, CTX-M-15	>64	4	1	>64	4	>64	0.5	>8	0.5
SR202170	Turkey	1284	YRIN, I532L	CMY-42, OXA-1, OXA-181, CTX-M-15	>64	4	2	>64	2	>64	0.5	>8	0.5
SR202171	Turkey	1284	YRIN, I532L	CMY-42, OXA-1, OXA-181, CTX-M-15	>64	32	4	>64	4	>64	4	>8	0.5

ATM, aztreonam; AVI, avibactam; CAZ, ceftazidime; COZ/TAZ, ceftolozane/tazobactam; FEP, cefepime; CFD, cefiderocol; MEM, meropenem; CIP, ciprofloxacin; CST, colistin. Avibactam and tazobactam were tested at a fixed concentration of 4 mg/L.

a Substitution and YRIN insertion (after P333) in PBP3 were compared with the sequence of *E. coli* MG1655 (K-12).

### Genetic backgrounds of the strains possessing the YRIN insertion

All nine strains from Turkey belonged to ST1284, whereas two strains from Italy and the USA belonged to ST361 (Table 1). Strains belonging to each sequence type displayed similar β-lactamase genotypes, with all nine strains from Turkey carrying CMY-42, OXA-1 and the OXA-181 carbapenemase (five strains additionally carried the CTX-M-15 ESBL), whereas the two strains from the USA and Italy carried CMY-42 and TEM-1 (Table 1). SNP analysis revealed that quite similar genetic backgrounds were shared among the nine strains collected in Turkey, with eight strains differing by only zero to four SNPs (Table S3). The two strains collected in Italy and the USA were less closely related (268 differences), but there were approximately 32 600 differences in SNPs between strains collected in Turkey and Italy/USA.

### Antimicrobial susceptibility of the strains possessing the YRIN insertion and isogenic mutants

All 11 sequenced strains showed reduced susceptibility to cefepime (MIC 4 to >64 mg/L) and ceftolozane/tazobactam (MIC 64 to >64 mg/L); the presence of a CTX-M-15 ESBL in 4 strains undoubtedly contributed to the high cefepime MIC of >64 mg/L that was observed (Table 1).^9^ In comparison, the strains remained susceptible (by CLSI breakpoints) to ceftazidime/avibactam and cefiderocol, with MICs ranging from 1 to 4 and 0.25 to 4 mg/L, respectively.^7^ The strains were susceptible to meropenem and colistin, with the exception of one strain, SR202171, that was meropenem resistant (MIC 4 mg/L), but all were resistant to ciprofloxacin (MIC >8 mg/L). Isogenic mutants possessing YIRN(K)-inserted PBP3 displayed ≥8-fold reduced susceptibility to several tested cephalosporins, including aztreonam and ceftazidime, and 4-fold reduced susceptibility to ceftazidime/avibactam (Table 2). No fold change was observed for meropenem.

**Table 2.** Antimicrobial susceptibility of isogenic *E. coli* strain possessing YRIN(K)-inserted PBP3

Antibacterial	MIC, mg/L (fold change)		
	*E*. *coli* MG1655	MG1655 PBP3::YRIN	MG1655 PBP3::YRIK
Aztreonam	0.12 (NA)	2 (16)	2 (16)
Cefepime	0.06 (NA)	0.5 (8)	0.5 (8)
Cefiderocol	0.06 (NA)	0.125 (2)	0.125 (2)
Ceftazidime	0.25 (NA)	2 (8)	2 (8)
Ceftazidime/avibactam	0.25 (NA)	1 (4)	1 (4)
Ceftolozane	0.25 (NA)	2 (8)	2 (8)
Ceftolozane/tazobactam	0.25 (NA)	2 (8)	2 (8)
Meropenem	0.03 (NA)	0.03 (1)	0.03 (1)

YRIN(K): four amino acid insertion after amino acid position P333. NA, not applicable.

## Discussion

All the identified strains of ST1284 lineage possessing the YRIN insertion from Turkey had a genetically clonal background and were collected from the same hospital, suggesting the occurrence of an outbreak limited to this hospital (Table S2). In addition, the two ST361 lineage strains also had a genetically clonal background, but these strains were isolated from distant countries, Italy and the USA. Although it is only possible to speculate how *E. coli* strains with YRIN-inserted PBP3 emerged and disseminated in each region, antibiotic pressure caused by inadequate treatment with cephalosporins or other β-lactam antibacterials may have contributed to the dissemination of these particular strains, which also carry several acquired β-lactamases. Strains of ST1284 and ST361 lineages possessing YRIN-inserted PBP3 were previously reported (including as part of NCBI database analysis), but the β-lactamases carried by these strains were consistent with the strains in the current study in only some cases.^1^^,^^3^ Among ST1284 strains possessing YRIN-inserted PBP3, one strain from India carried CMY-42, OXA-1 and CTX-M-15, but another strain from Lebanon carried CTX-M-15. Among ST361 strains possessing YRIN-inserted PBP3, one strain from Kuwait carried CMY-42 and TEM-1, as observed in this study, but seven other ST361 strains from China carried NDM-5 and CTX-M-55.^1^^,^^3^ These data suggest that at least strains of ST1284 and ST361 lineages, possessing YRIN-inserted PBP3, are disseminating among several regions, possibly with stepwise acquisition of β-lactam resistance. The YRIN insertion in PBP3 occurred with multiple β-lactamases, which indicates frequent cross-resistance to other β-lactams.

The YRIN insertion in PBP3 is predicted to have a negative impact on the antibacterial activity of several β-lactam antibacterials, such as cephalosporins and monobactams, that mainly target *E. coli* PBP3.^1^ In addition, since the identified *E. coli* strains possessing YRIN-inserted PBP3 also acquired multiple β-lactamases, including carbapenemases, the resulting cross-resistance and lack of treatment options would be problematic in the clinical setting. Indeed, in a study of isogenic mutants, the presence of the YRIN insertion appeared to impact susceptibility to several well-used antibacterials. Regarding ceftazidime/avibactam, the MICs of this agent against clinical strains possessing YRIN-inserted PBP3 remained susceptible but were relatively higher (1–4 mg/L) than the MICs obtained against all 1529 *E. coli* isolates in the SIDERO-WT-2014 study (MIC_50_ and MIC_90_ 0.12 and 0.25 mg/L, respectively).^4^ There were only 21 strains among 1529 *E. coli* isolates that showed a ceftazidime/avibactam MIC of ≥1 mg/L, including 11 strains possessing YRIN-inserted PBP3 and one VIM-type metallo-β-lactamase producer (Y. Yamano, Shionogi, unpublished data). Therefore, a four amino acid insertion in PBP3 might be suspected in clinical *E. coli* isolates with ceftazidime/avibactam MIC of ≥1 mg/L that do not carry metallo-β-lactamases.

In summary, we analysed the characteristics of *E. coli* possessing PBP3 substitutions that affect cephalosporin susceptibility using the clinical isolates collected as part of the SIDERO-WT-2014 surveillance study. The similarities in the genetic backgrounds of the strains identified in our study and strains reported by others suggest that some clonal strains of the ST1284 and ST361 lineages have disseminated among multiple regions. Although they remained susceptible to several antibacterials, these strains need to be carefully monitored because of cross-resistance to β-lactam antibacterials caused by the YRIN insertion as well as acquired β-lactamases.

### Limitations

Because the *ftsI* gene was only sequenced in a subset of *E. coli* isolates with MICs ≥2 mg/L for one of three PBP3-targeted cephalosporins, the true prevalence of isolates possessing four amino acid insertions in PBP3 cannot be determined for the SIDERO-WT-2014 surveillance collection.

## Supplementary Material

dlaa081_Supplementary_Data
